# Incidence and mortality from breast and cervical cancer in a Brazilian town

**DOI:** 10.11606/s1518-8787.2021055003085

**Published:** 2021-10-18

**Authors:** Maria do Carmo Ferreira, Diama Bhadra Vale, Marilisa Berti de Azevedo Barros

**Affiliations:** I Universidade Estadual de Campinas Faculdade de Ciências Médicas Programa de Pós-Graduação em Saúde Coletiva CampinasSP Brasil Universidade Estadual de Campinas. Faculdade de Ciências Médicas. Programa de Pós-Graduação em Saúde Coletiva. Campinas, SP, Brasil; II Universidade Estadual de Campinas Faculdade de Ciências Médicas Departamento de Tocoginecologia CampinasSP Brasil Universidade Estadual de Campinas. Faculdade de Ciências Médicas. Departamento de Tocoginecologia. Campinas, SP, Brasil; III Universidade Estadual de Campinas Faculdade de Ciências Médicas Departamento de Saúde Coletiva CampinasSP Brasil Universidade Estadual de Campinas. Faculdade de Ciências Médicas. Departamento de Saúde Coletiva. Campinas, SP, Brasil

**Keywords:** Breast Neoplasms, epidemiology. Uterine Cervical Neoplasms, epidemiology. Mortality, trends

## Abstract

**OBJECTIVES:**

To analyze the magnitude of changes in the incidence and mortality from cervical cancer (CC) and breast cancer (BC) in Campinas, São Paulo State, between the five-year periods of 1991–1995 and 2010–2014.

**METHODS:**

data on cancer were obtained from the Campinas Population-Based Cancer Registry and data on deaths from the Mortality Information System of the Computing Department of the Unified Health System. Age-standardized incidence and mortality rates were calculated by the direct method, with the respective 95% confidence intervals. The magnitude of the changes was measured by the rate ratio (rate ratio; 95%CI).

**RESULTS:**

among the periods studied, there was a threefold increase in the detection rate of *in situ* CC (3.03; 95%CI: 2.64–3.47) and fivefold increase for *in situ* BC (5.23; 95%CI: 4.98–5.50). The proportion of cases of *in situ* BC in relation to the total number of cases of BC increased from 3.31% to 11.05%. The incidence rate of invasive CC decreased by 57% (0.43; 95%CI: 0.40–0.47), and the incidence rate of invasive BC increased by 40% (1.40; 95%CI: 1.33–1.47). The mortality rate of the CC decreased by 58% (0.42; 95%CI: 0.32–0.56), and that of BC by 15% (0.85; 95%CI: 0.82–0.89).

**CONCLUSION:**

the incidence of *in situ* carcinomas of CC and BC increased in almost two decades. The rate of invasive carcinoma of CC decreased, and that of BC increased. Mortality from both cancers decreased. Observing these changes is useful for assessing the impact of the actions carried out in the period and for planning future actions.

## INTRODUCTION

Breast and cervical cancer are prominent causes of female morbidity and mortality worldwide. Excluding non-melanoma skin neoplasms, breast cancer is the most common among women, accounting for 2.1 million new cases and approximately 600 thousand deaths in 2018^1^. In Brazil, this is the most incident cancer in females in all regions of the country^2^.

With lower incidence, but still high mortality, cervical cancer is the fourth most frequent among women in the world, being responsible for 510 thousand new cases and 311 thousand deaths in 2018. The highest incidence rates occur in Sub-Saharan African and Southeast Asian countries^1^. In Brazil, it is the second most frequent cancer among women in the North, Northeast and Midwest regions, while in the South and Southeast regions it is the fourth and fifth most frequent, respectively^2^.

Since the 2000s, incidence and mortality from breast cancer have fallen in developed countries, but in developing countries both incidence and mortality have shown an increasing tendency^1,3^. In recent decades, there has also been a decline in cervical cancer incidence and mortality worldwide, although some countries in Central Europe, Eastern Europe and Asia have seen increases among young women. This increase, observed since de 1990s, is attributed to a growing incidence of infection by the human papilomavírus (HPV)^1,4^.

In Brazil, the Ministry of Health has had guidelines for early detection of breast and cervical cancers since the 1980s. In 1984, the Women’s Health Comprehensive Care Program recommended Pap smears in routine gynecological consultations, in addition to clinical examination and breast self-examination. In 1986, the Oncology Program devised the project to expand prevention and control of cervical cancer, which aimed to integrate existing programs and articulate the service network on different levels of care. In the mid-1990s, with the Unified Health System (SUS) already created, the *Viva Mulher* program was elaborated to control cervical cancer. In 1999, other prevention and control actions were incorporated, but only in 2003 was the process strengthened. In 2005, the National Cancer Care Policy set goals to be met by federal agencies to control these cancers^5^.

The expansion of primary care in the last 30 years, from 19.000 primary healthcare units (UBS) in 1988 to more than 41.000 in 2010, improved access for the population^8^. Primary care organizes the system and executes actions to promote health, prevention and early diagnosis, including screening for cervical cancer with Pap smears and requesting mammography to detect breast cancer. Positive cases are managed by the referred specialized attention. The ultimate goal is to reduce the incidence and mortality from these cancers in the country^9^. Improving survival rates is one of the expected intermediate outcomes.

Trend analyses of cancer incidence in Brazil are scarce because they depend on active population-based cancer records, which need to be sufficient in length to allow a perception of changes over time^10^. However, in 2015 the reactivation of the Population-Based Cancer Registry by the City Health Department of Campinas made it possible to assess changes that occurred in the town between two periods separated by two decades.

Considering these data, the objectives of this study are to analyze the magnitude of changes in incidence and mortality from cervical and breast cancer between the five-year periods 1991–1995 and 2010–2014; and to verify overdiagnosis in the incidence of breast cancer in the two periods analyzed, which correspond to a period of consolidation of the SUS and implementation of screening protocols for the neoplasms studied.

## METHODS

This is a population-based study that analyzes incidence and mortality data from breast cancer and cervical cancer among women living in Campinas. The analyses refer to the periods of 1991–1995 and 2010–2014.

Campinas is 99 kilometers northwest of the state capital of São Paulo. It is the 14^th^ most populous city in the country, with 1,213,792 inhabitants according to 2020 data (IBGE)^14^. The town is the https://cidades.ibge.gov.br/brasil/sp/campinas/panorama seat of the Campinas Metropolitan Area, in which it is a reference for cancer treatment in both public and private sectors.

For the period of 1991–1995, the source of data on cancer cases was the National Cancer Institute (INCA), which provided data from the period originally belonging to the Campinas Population-Based Cancer Registry (RCBP). For the period of 2010–2014, the data were obtained directly from the Campinas RCBP, which, when reactivated by the city’s Health Department in 2015, collected data of cases with diagnosis starting in 2010. The standardization of collection and registration of new cases of *in situ* and invasive cancer, identical for both periods, followed the recommendations of the International Agency for Research on Cancer (IARC)^15^. The source of data on deaths in both periods was the Mortality Information System of the Unified Health System’s Computing Department. Population data were obtained from the Brazilian Institute of Geography and Statistics.

Cancer cases and deaths of the first period were coded by the Ninth Revision of the International Classification of Diseases (ICD-9): *in situ* cervical cancer (CC) (233.0/1), invasive CC (180), *in situ* breast cancer (BC) (233.0/2) and invasive BC (174). In the second period, for coding cases and deaths, the Tenth Revision of the International Classification of diseases (ICD-10) was used, with the respective codes: *in situ* CC (D06), invasive CC (C53), *in situ* BC (D05) and invasive BC (C50).

**Figure 1:**
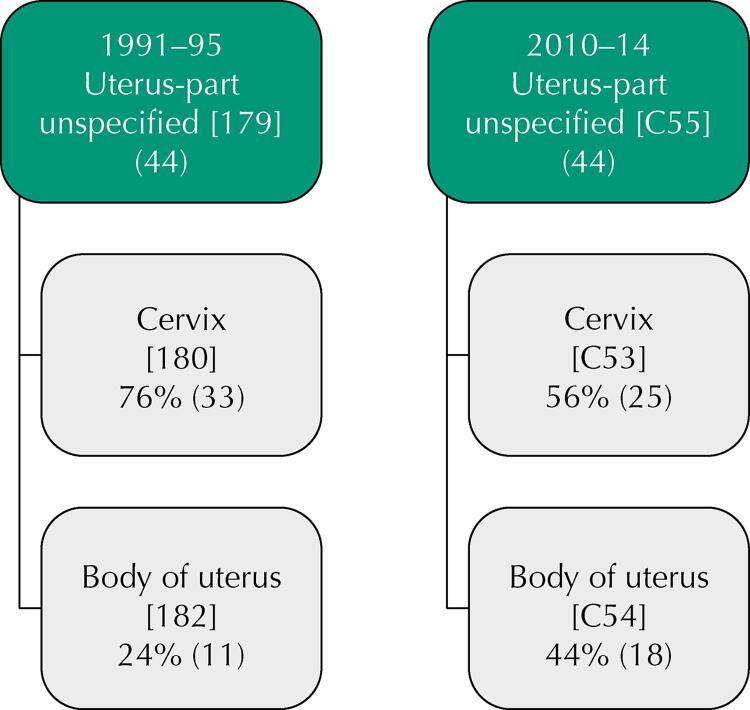
Relocation of cervical cancer deaths from the unspecified portion (SOE) to the cervix and body of the uterus.

Adapted from Loos et al.^16^

Deaths from CC were corrected by proportionally relocating cancer deaths from “uterus, part unspecified – SOE”, coded as 179 (1991–1995) and C55 (2010–2014) to cervix (180 and C53) and body of uterus (182 and C54), respectively^16^ (Figure 1). With this correction, 58 deaths were added to CC, 33 in the first period and 25 in the second. The relocation considered distribution by age groups. To assess overdiagnosis among newly diagnosed cases of BC in the periods studied, the study calculated the proportion of *in situ* BC in relation to the total BC.

For each five-year period, the study calculated the mean values of incidence rates of cervical and breast cancers, *in situ* and invasive, and mortality rates of both types of cancer. The crude mortality and incidence rates per 100 thousand women were also calculated, and then the age-standardized rates were estimated by the direct method, using the 1960 world standard population, modified by Doll et al.^17^ in 1966, used in INCA publications. For the crude and standardized rates, the ratios between the rates (RT) were estimated. To calculate the confidence intervals of 95% of the ratios between standardized rates, the formula was used $IC=\left(\frac{TPI^{1}}{TPI^{2}}\right)1\pm \left(\frac{1.96}{X}\right)$, where $X={\frac{TPI^{1}+TPI^{2}}{\sqrt{EP{\left(TPI^{1}\right)}^{2}+EP{\left(TPI^{2}\right)}^{2}}}}_{15}$^15^. A significance level of 5% was adopted. Statistical analyses were performed in Microsoft Excel 2016.

The research project that resulted in this article was approved by the Ethics Committee of *Faculdade de Ciências Médicas da Universidade Estadual de Campinas* (CAAE: 09217719.9.0000.5404).

## RESULTS

Between 1991 and 1995, in the town of Campinas, invasive CC was the third most common cancer in women, accounting for 9.3% of malignant neoplasms in women if non-melanoma skin neoplasms are excluded. Between 2010 and 2014, invasive CC ranked ninth among malignant neoplasms in females, accounting for only 3% of cancer cases among women.

Between 1991–1995 and 2010–2014, the age–standardized detection rate of *in situ* cervical cancer increased from 5.32 to 16.11 per 100 thousand women, with a rate ratio of 3.03 (95% CI 2.64–3.47). The standardized incidence rate of invasive cervical cancer, in turn, decreased from 16.08 to 6.94 cases per 100 thousand women, resulting in a rate ratio of 0.43 (95% CI 0.40–0.47). The mortality rate also fell significantly between the two periods, from 6.97 to 2.95 deaths per 100 thousand women, resulting in a rate ratio of 0.42 (CI 95% 0.32–0.56) (Table 1).

**Table 1:** Incidence and mortality of cervical cancer (rates per 100 thousand women), Campinas, 1991–1995 and 2010–2014.

Rate	1991–1995 (1)	2010–2014 (2)	Ratio between rates (2/1)
Crude detection rate of *in situ* cancer	5.83 (4.83–6.83)^a^	19.53 (17.91–21.15)^a^	3.35
Standardized rate^b^ of *in situ* cancer detection	5.32 (4.38–6.26)^a^	16.11 (14.77–17.46)^a^	3,03 (2.64–3.47)^a^
Crude incidence rate of invasive cancer	14.61 (13.03–16.20)^a^	8.87 (7.78–9.96)^a^	0.61
Standardized rate^b^ of incidence of invasive cancer	16.08 (14.31–17.86)^a^	6.94 (6.07–7.82)^a^	0.43 (0.40–0.47)^a^
Crude mortality rate	6.14 (5.11–7.17)^a^	3.86 (3.14–4.58)^a^	0.63
Standardized mortality rate^b^	6.97 (5.79–8.15)^a^	2.95 (2.39–3.51)^a^	0.42 (0.32–0.56)^a^

^a^ 95% confidence interval.

^b^ Standardized rates by age by the 1960 world standard population, modified by Doll et al^17^.

Breast cancer, excluding non-melanoma skin neoplasms, was already the most incident between 1991 and 1995, accounting for 28.9% of female cancers, a similar percentage to the one observed between 2010 and 2014 (29.6%).

The age-standardized detection rate of *in situ* breast carcinoma increased from 1.70 in the first period to 8.91 per 100 thousand women in the second, reaching a rate ratio of 5.23 (CI 95% 4.98–5.50). The incidence rate of invasive tumor also increased from 50.15 to 70.03 per 100 thousand women, with a rate ratio of 1.40 (CI 95% 1.33–1.47), while the mortality rate decreased between the periods analyzed, going from 16.18 to 13.79 per 100 thousand women, with a rate ratio of 0.85 (CI 95% 0.82–0.89) (Table 2).

**Table 2:** Incidence and mortality of breast cancer (rates per 100 thousand women), Campinas, 1991–1995 and 2010–2014.

Rate	1991–1995 (1)	2010 –2014 (2)	Ratio between rates (2/1)
Crude detection rate of *in situ* cancer	1.52 (1.01–2.04)^a^	11.04 (9.82–12.26)^a^	7.25
Standardized rate^b^ of *in situ* cancer detection	1.70 (1.16–2.24)^a^	8.91 (8.61–9.21)^a^	5.23 (4.98–5.50)^a^
Crude incidence rate of invasive cancer	44.51 (41.74–47.28)^a^	88.92 (85,44–92,38)^a^	2.01
Standardized rate^b^ of incidence of invasive cancer	50.15 (46.98–53.31)^a^	70.03 (67.27–72.79)^a^	1.40 (1.33–1.47)^a^
Crude mortality rate	13.94 (12.39–15.49)^a^	18.30 (16.73–19.87)^a^	1.31
Standardized mortality rate^b^	16.18 (15.67–16.69)^a^	13.79 (13.46–14.12)^a^	0.85 (0.82–0.89)^a^

^a^ 95% confidence interval.

^b^ Standardized rates by age by the 1960 world standard population, modified by Doll et al^17^.

The proportion of cases of *in situ* BC in relation to the total breast cancers diagnosed was 3.31% in the period of 1991–1995 and 11.05% in the period of 2010–2014.

## DISCUSSION

The results of this study showed, between the periods analyzed, a threefold increase in the detection rate of *in situ* CC and a 5.2 time increase for *in situ* BC, as well as a decrease in mortality rates for both BC (15% decrease) and CC (58%). However, while the rate of invasive tumor decreases significantly (57%) for CC, a 40% increase is observed for invasive BC.

Decreasing incidence and mortality rates of CC have been observed in most countries in recent decades^3,4^. Analysis with data from the RCBP of ten Brazilian capital cities in different periods, from 1996 to 2011, showed a decrease in the incidence of invasive CC in most cities, especially in São Paulo, Goiânia and Curitiba, while *in situ* CC showed an increasing trend in all places studied^12^. In the town of Aracaju, in the period of 1996–2015, the incidence rate of *in situ* CC went from 1.19 to 30.2 per 100 thousand women, while that of invasive CC decreased from 32.3 to 9.1 per 100 thousand women^13^. A study conducted in the Barretos region (São Paulo State) detected, between 2000 and 2015, an important increase in the incidence of *in situ* CC, which went from 10.9 to 24.84 per 100 thousand women, while the incidence of invasive CC remained stable^11^.

The reduction in CC mortality found in Campinas was also observed in other studies conducted in Brazil, but trends differ between regions and states in different periods^18,19^. A study that analyzed the trend of CC mortality in Brazil in three decades, from 1980 to 2010, showed a decrease in the entire period, except in the towns in the countryside of Northern and Northeastern states^18^. This decrease probably reflects the expansion of access to Pap smear, which may not have occurred in countryside towns the North and Northeast regions. Analyzing the trend of mortality from CC between 2003 and 2012, Vale et al. found that the rates did not decrease only in the North region, and that the largest drops were found in the Midwest and South regions^19^. A significant reduction in mortality from CC (36%) was also observed in the town of Aracajú, where the mortality rate fell from 8.1 in 1996 to 5.2 per 100,000 women in 2015^13^.

Regarding breast cancer, in Campinas the detection of *in situ* BC (524%) and the incidence rate of invasive BC (40%) increased between the periods analyzed. This increasing trend was also observed in the Barretos region between 2000 and 2015, with the incidence rate of *in situ* tumor increasing from 1.6 to 16, and the that of invasive BC from 29.1 to 64.1 per 100 thousand women^11^.

The increased incidence of BC found in Campinas and in the Barretos region also occurs worldwide. The largest increases in recent years have been detected in countries in South America, Africa and Asia^1^. In Europe, North America and Australia, the incidence increased in the 1980s until the mid-1990s, when it stabilized, and began to decrease in the 2000s^1,3^. The causes of the increase in the 1980s were: improved breast cancer screening, increased prevalence of lifestyle-related risk factors and hormone replacement therapy^3,20^. No uniform trend in BC incidence has been found in Brazil. Capitals such as Aracaju, Goiânia and Belo Horizonte have shown an increasing trend in recent decades, while São Paulo and Curitiba show a decreasing trend^21^.

The mortality rate from BC in Campinas did not follow the increase in incidence, instead showing a decrease between the periods studied, unlike what happened in other places in Brazil, but in line with what was observed in towns in Southern and Southeastern Brazil^18,21^. Mortality from breast cancer begins to fall in the 1990s in the capitals of the South and Southeast regions, but increases in the other regions^21^. The difference must be related to access to mammograms and increased incidence in some regions.

The increased detection of *in situ* CC in Campinas is probably due to the screening strategy implemented in the town in the 1960s^22^. On the other hand, the reduced incidence of invasive CC can be attributed to an increase in the diagnoses of *in situ* CC, an available care network for treatment of precursor lesions and patient follow-up. The Campinas region was a pioneer in implementing cytologic examination for CC diagnosis through a partnership between Universidade Estadual de Campinas and the city and state health secretariats. Decentralized oncotic cytology collection, which began in UBS, expanded women’s access to early CC diagnosis. The decrease in mortality during the study period must be related to the decrease in the incidence of invasive CC and the increase in detection at earlier stages, allowing more timely treatments that increase the potential for cure^23^.

The increased detection of *in situ* BC may be a consequence of better access to mammograms^24,25^. It must be considered, however, that such an increase may reflect some degree of overdiagnosis, that is, the detection of indolent tumors through mammograms that would not manifest clinically. Excess incidence calculations based on large-scale program audits estimate that around 20% to 50% of *in situ* or invasive cases detected through screening may represent overdiagnosis^26^. It is desirable that the proportion of *in situ* cases be up to 15% of total diagnosed turmors^27^. In this study, the number of *in situ* BC corresponded to 3.31% in the period of 1991–1995 and 11.05% in the period of 2010–2014, therefore, a above the range considered as an indicator of overdiagnosis.

The increased incidence rate of invasive BC between the periods analyzed may reflect greater exposure of women to risk factors linked to life habits, such as obesity, first pregnancy after age 30, fewer children and a sedentary lifestyle, among others, as well as better access to hormone replacement therapy^20^. Diagnosis at earlier stages and better treatments may have contributed to the decrease in mortality from BC observed in this study^25^.

The coverage of Pap smear and mammography examination in Campinas has increased in recent years. Data from the Campinas Population-Based Health Survey (ISACAMP) show an increase in Pap smear coverage among women aged 40 to 59 years, from 80.6% to 87.8% between 2002 and 2008^24,28^. Mammograms among women aged 40 years or older in the two years prior to the interview increased from 52.1% in 2002 to 64.2% in 2008^24^. It is noteworthy that these results refer to mammograms in the last two years and Pap smear in the last three years based on self-reported information provided by the interviewees.

A study that analyzed information from Hospital Cancer Records in São Paulo State between 2000 and 2015 showed an increase in the diagnosis of localized breast tumors and in the early stages of the disease, which may explain the increase in the incidence of *in situ* and invasive BC and the decrease in breast cancer mortality reported in Campinas^25^.

The municipality of Campinas has had a structured primary health network since the 1970s. This network has been expanded over the last decades and currently has 66 UBS. Primary care, which structures assistance and coordinates care, plays a key role in delivering, preventing and planning care for suspected and diagnosed cases of cancer in the territory it covers^9^. In 2015, Campinas had primary care coverage of approximately 50%. In the following years, this coverage decreased, and in 2019 the town had 128 family health teams and covered 36.5% of the population^29^.

It is necessary to consider that this study uses data from the first years of the Campinas RCBP, in its two implementation stages, 1991 and 2010. However, the RCBP information for the period from 1991 to 1995 was part of the publication of the (body of the World Health Organization) in the document *Cancer incidence in five continents: volume VIII*^30^, published in 2002, which attests to its quality. And in the period from 2010 to 2014, the coverage indicators of the RCBP are in accordance with IARC recommendations^31^.

There are few studies on cancer incidence in Brazil since data are available only for towns that have an active RCBP. In 2019, there were 33 RCBP in Brazil, of which only 27 were active^2^. Furthermore, it is necessary that data cover a long period of time for changes to be analyzed, which was possible in this study since the two periods considered are separated by 20 years.

For screening programs to be effective, it is important to organize the health network, both in primary care and in reference units for treatment. The strategic planning of the Ministry of Health for the 2011–2015 period set forth actions to be taken by the Federal Government to qualify the Mammography Program and expand diagnostic services of BC and CCU^32^. Nevertheless, besides ensuring access to preventive practices (which was made possible by the significant growth and strengthening of primary care), it is also essential that treatment be delivered in a timely manner.

## CONCLUSIONS

In almost two decades, the incidence rates of *in situ* BC and CC carcinomas have increased significantly (more than three and seven times, respectively). The rate of invasive CC carcinoma decreased, but that of BC increased. As a positive result, this study found a decrease in the mortality rates from BC and the persistent decrease of deaths from CC. Observing these changes helps to assess the impact of actions taken in the period and to plan future actions.
