# Supplementary material for: Incidence and mortality from breast and cervical cancer in a Brazilian town
Source: Rev Saude Publica. 2021 Oct 18;55:67. doi: 10.11606/s1518-8787.2021055003085 (PMC8522755; doi:10.11606/s1518-8787.2021055003085)
Supplement: Translation

# Translation: Incidência e mortalidade por câncer de mama e do colo do útero em um município brasileiro

**Authors:** Maria do Carmo Ferreira, Diama Bhadra Vale, Marilisa Berti de Azevedo Barros

**Affiliations:** I Brasil Universidade Estadual de Campinas. Faculdade de Ciências Médicas. Programa de Pós-Graduação em Saúde Coletiva. Campinas, SP, Brasil; II Brasil Universidade Estadual de Campinas. Faculdade de Ciências Médicas. Departamento de Tocoginecologia. Campinas, SP, Brasil; III Brasil Universidade Estadual de Campinas. Faculdade de Ciências Médicas. Departamento de Saúde Coletiva. Campinas, SP, Brasil

**Keywords:** Neoplasias da Mama, Epidemiologia. Neoplasias do Colo do Útero, Epidemiologia. Mortalidade, Tendências

## Abstract

**OBJETIVOS:**

Analisar a magnitude das mudanças na incidência e mortalidade por câncer do colo do útero (CCU) e câncer de mama (CM) em Campinas (SP) entre os quinquênios 1991–1995 e 2010–2014.

**MÉTODOS:**

Os dados sobre câncer foram obtidos do Registro de Câncer de Base Populacional de Campinas e os dados sobre óbitos do Sistema de Informação de Mortalidade do Departamento de Informática do Sistema Único de Saúde. Foram calculadas taxas de incidência e de mortalidade padronizadas por idade pelo método direto, com os respectivos intervalos de confiança de 95%. Mensurou-se a magnitude das mudanças por meio da razão de taxas (RT).

**RESULTADOS:**

Entre os períodos estudados, houve aumento de três vezes na taxa de detecção do CCU *in situ* (RT = 3,03; IC95% 2,64–3,47) e de 5 vezes para o CM *in situ* (RT = 5,23; IC95% 4,98–5,50). A proporção de casos de CM *in situ* em relação ao total de casos de CM aumentou de 3,31% para 11,05%. A taxa de incidência do CCU invasivo declinou em 57% (RT = 0,43; IC95% 0,40–0,47), e a do CM invasivo aumentou em 40% (RT = 1,40; IC95% 1,33–1,47). A taxa de mortalidade do CCU caiu em 58% (RT = 0,42; IC95% 0,32–0,56), e a do CM em 15% (RT = 0,85; IC95% 0,82–0,89).

**CONCLUSÃO:**

A incidência de carcinomas *in situ* de CCU e CM aumentou nessas quase duas décadas. A taxa de carcinoma invasivo do CCU diminuiu, e a do CM aumentou. A mortalidade pelos dois cânceres foi reduzida. Observar essas alterações é útil para avaliar o impacto das ações realizadas no período e planejar ações futuras.

## INTRODUÇÃO

O câncer de mama e do colo do útero são causas proeminentes de morbimortalidade feminina em todo o mundo. Excluindo as neoplasias de pele não melanoma, o câncer de mama é o mais comum entre mulheres, contabilizando 2,1 milhões de casos novos e aproximadamente 600 mil mortes em 2018^1^. No Brasil, trata-se do câncer mais incidente no sexo feminino em todas as regiões do país^2^.

Com incidência menor, mas ainda alta mortalidade, o câncer do colo do útero é o quarto mais frequente entre as mulheres no mundo, tendo sido responsável por 510 mil novos casos e 311 mil óbitos em 2018. As mais altas incidências ocorrem nos países da África Subsaariana e do Sudeste Asiático^1^. No Brasil, trata-se do segundo câncer mais frequente entre as mulheres das regiões Norte, Nordeste e Centro-Oeste, enquanto nas regiões Sul e Sudeste ocupa a quarta e a quinta posição, respectivamente^2^.

Desde os anos 2000, vem se observando queda da incidência e da mortalidade por câncer de mama em países desenvolvidos, mas nos países em desenvolvimento tanto a incidência quanto a mortalidade apresentam tendência de aumento^1,3^. Nas últimas décadas, também tem-se observado declínio da incidência e da mortalidade por câncer do colo do útero em todo o mundo, embora alguns países da Europa Central, do Leste Europeu e da Ásia venham registrando aumento entre mulheres jovens. Esse aumento, observado desde o final da década de noventa, é atribuído ao crescimento da incidência da infecção pelo papilomavírus humano (HPV)^1,4^.

No Brasil, o Ministério da Saúde tem diretrizes voltadas à detecção precoce dos cânceres de mama e do colo do útero desde a década de 1980. Em 1984, o Programa de Atenção Integral à Saúde da Mulher previa a coleta do Papanicolaou nas consultas ginecológicas de rotina, assim como exame clínico e autoexame das mamas. Em 1986, o Programa de Oncologia elaborou o Projeto de Expansão da Prevenção e Controle do Câncer Cérvico-Uterino, que visava integrar os programas existentes e articular a rede de serviços nos diferentes níveis de atenção. Em meados da década de 1990, com o Sistema Único de Saúde (SUS) já criado, foi elaborado o programa Viva Mulher, para controle do câncer do colo do útero. Em 1999, outras ações de prevenção e controle foram incorporadas, mas somente em 2003 o processo se fortaleceu. Em 2005, a Política Nacional de Atenção Oncológica estabeleceu metas, a serem cumpridas pelos entes federados, para o controle desses cânceres^5^.

A ampliação da atenção básica nos últimos 30 anos, passando de 19 mil Unidades Básicas de Saúde (UBS) em 1988 para mais de 41 mil em 2010, favoreceu o acesso da população^8^. A atenção básica é a ordenadora do sistema e executora de ações de promoção da saúde, prevenção e diagnóstico precoce, incluindo o rastreamento do câncer do colo do útero por meio do teste de Papanicolaou e da solicitação de exame de mamografia para detecção do câncer de mama. O manejo dos casos positivos é realizado pela atenção especializada referenciada. O objetivo final é reduzir a incidência e a mortalidade por esses tipos de câncer no país^9^. Melhorar as taxas de sobrevida é um dos resultados intermediários esperados.

Análises de tendência da incidência de câncer no Brasil são escassas pois dependem de registros de câncer de base populacional ativos, com duração suficiente para possibilitar a percepção de mudanças no decorrer do tempo^10^. Entretanto, em 2015, a reativação do Registro de Câncer de Base Populacional pela Secretaria Municipal de Saúde de Campinas tornou possível avaliar mudanças ocorridas no município entre dois períodos distanciados por duas décadas.

Considerando esses dados, os objetivos do presente estudo são analisar a magnitude das mudanças na incidência e na mortalidade por câncer do colo do útero e câncer de mama entre os quinquênios 1991–1995 e 2010–2014; e verificar o sobrediagnóstico na incidência do câncer de mama nos dois períodos analisados, que correspondem a um período de consolidação do SUS e implementação de protocolos de rastreamento para as neoplasias estudadas.

## MÉTODOS

Este é um estudo de base populacional que analisa dados de incidência e mortalidade por câncer de mama e do colo do útero entre mulheres residentes em Campinas. As análises se referem aos períodos de 1991–1995 e 2010–2014.

Campinas se situa a 99 quilômetros a noroeste da capital do estado de São Paulo e é o 14º município mais populoso do país, com 1.213.792 habitantes segundo dados de 2020 (IBGE)^14^. O município é sede da Região Metropolitana de Campinas, dentro da qual é referência no tratamento de câncer tanto no setor público quanto privado.

Para o período 1991–1995, a fonte dos dados de casos de câncer foi o Instituto Nacional do Câncer (Inca), que forneceu dados da época originalmente pertencentes ao Registro de Câncer de Base Populacional (RCBP) de Campinas. Para o período 2010–2014, os dados foram obtidos diretamente do RCBP de Campinas, que, quando reativado pela Secretaria Municipal de Saúde em 2015, coletou dados de casos com diagnóstico a partir de 2010. A padronização da coleta e registro de casos novos de câncer *in situ* e invasivo, idêntica para os dois períodos, seguiu as recomendações da Agência Internacional de Pesquisa em Câncer (Iarc)^15^. A fonte de dados dos óbitos de ambos os períodos foi o Sistema de Informação de Mortalidade do Departamento de Informática do Sistema Único de Saúde. Os dados de população foram obtidos do Instituto Brasileiro de Geografia e Estatística.

Os casos de câncer e óbitos do primeiro período foram codificados pela Nona Revisão da Classificação Internacional de Doenças (CID-9): câncer do colo do útero (CCU) *in situ* (233.0/1), CCU invasivo (180), câncer de mama (CM) *in situ* (233.0/2) e CM invasivo (174). No segundo período, para a codificação dos casos e óbitos, foi utilizada a Décima Revisão da Classificação Internacional de Doenças (CID-10), com os respectivos códigos: CCU *in situ* (D06), CCU invasivo (C53), CM *in situ* (D05) e CM invasivo (C50).

Foi realizada a correção dos óbitos por CCU, realocando proporcionalmente os óbitos por câncer de “útero, porção não especificada- SOE”, codificados como 179 (1991–1995) e C55 (2010–2014) para colo do útero (180 e C53) e corpo do útero (182 e C54), respectivamente^16^(Figura 1). Com essa correção, foram somados para CCU 58 óbitos, 33 no primeiro período e 25 no segundo. A realocação considerou a distribuição por faixas etárias. Para avaliar o sobrediagnóstico entre os casos novos diagnosticados de CM nos períodos estudados, foi calculada a proporção de CM *in situ* em relação ao total dos CM.

**Figura 1:** Realocação dos óbitos por câncer do colo do útero da porção não especificada (SOE) para colo do útero e corpo do útero.

Adaptado de Loos et al.^16^

Para cada quinquênio, foram calculados os valores médios das taxas de incidência dos cânceres do colo do útero e de mama, *in situ* e invasivo, e das taxas de mortalidade dos dois tipos de câncer. As taxas brutas de mortalidade e de incidência por 100 mil mulheres também foram calculadas, e a seguir foram estimadas as taxas padronizadas por idade pelo método direto, utilizando a população-padrão mundial de 1960 modificada por Doll et al.^17^ em 1966, utilizada nas publicações do Inca. Para as taxas brutas e padronizadas, foram estimadas as razões entre as taxas (RT). Para calcular os intervalos de confiança de 95% das razões entre taxas padronizadas, utilizou-se a fórmula $IC=\left(\frac{TPI^{1}}{TPI^{2}}\right)1\pm \left(\frac{1.96}{X}\right)$, onde $X\;=\;\frac{TPI^{1}\;+\;TPI^{2}\;}{{\sqrt{EP\;(TPI^{1}{)}^{2}\;+\;EP\;(TPI^{2}{)}^{2}\;}}^{15}}$^15^. Adotou-se um nível de significância de 5%. As análises estatísticas foram realizadas no software Microsoft Excel 2016.

O projeto de pesquisa que resultou neste artigo foi aprovado pela Comissão de Ética da Faculdade de Ciências Médicas da Universidade Estadual de Campinas (CAAE: 09217719.9.0000.5404).

## RESULTADOS

Entre 1991 e 1995, no município de Campinas, o CCU invasivo era o terceiro câncer mais incidente no sexo feminino, respondendo por 9,3% das neoplasias malignas em mulheres se excluídas as neoplasias de pele não melanoma. Entre 2010 e 2014, o CCU invasivo passa a ocupar a nona posição entre as neoplasias malignas no sexo feminino, respondendo por apenas 3% dos casos de câncer entre as mulheres.

Entre 1991–1995 e 2010–2014, a taxa de detecção padronizada por idade do câncer do colo do útero *in situ* aumentou de 5,32 para 16,11 por 100 mil mulheres, com uma razão de taxas de 3,03 (IC95% 2,64–3,47). A taxa de incidência padronizada do câncer do colo do útero invasivo, por sua vez, declinou de 16,08 para 6,94 casos por 100 mil mulheres, resultando numa razão de taxas de 0,43 (IC95% 0,40–0,47). A taxa de mortalidade também teve declínio importante entre os dois períodos, indo de 6,97 para 2,95 óbitos por 100 mil mulheres, resultando numa razão de taxas de 0,42 (IC95% 0,32–0,56) (Tabela 1).

**Tabela 1:** Incidência e mortalidade de câncer do colo de útero (taxas por 100 mil mulheres), Campinas, 1991–1995 e 2010–2014.

Taxas	1991–1995 (1)	2010–2014 (2)	Razão entre taxas (2/1)
Taxa bruta de detecção do câncer *in situ*	5,83 (4,83–6,83)^a^	19,53 (17,91–21,15)^a^	3,35
Taxa padronizada^b^ de detecção do câncer *in situ*	5,32 (4,38–6,26)^a^	16,11 (14,77–17,46)^a^	3,03 (2,64–3,47)^a^
Taxa bruta de incidência do câncer invasivo	14,61 (13,03–16,20)^a^	8,87 (7,78–9,96)^a^	0,61
Taxa padronizada^b^ de incidência do câncer invasivo	16,08 (14,31–17,86)^a^	6,94 (6,07–7,82)^a^	0,43 (0,40–0,47)^a^
Taxa bruta de mortalidade	6,14 (5,11–7,17)^a^	3,86 (3,14–4,58)^a^	0,63
Taxa padronizada^b^ de mortalidade	6,97 (5,79–8,15)^a^	2,95 (2,39–3,51)^a^	0,42 (0,32–0,56)^a^

^a^ Intervalo de confiança de 95%.

^b^ Taxas padronizadas por idade pela população-padrão mundial de 1960, modificada por Doll et al^17^.

O câncer de mama, excluídas as neoplasias de pele não melanoma, já era o mais incidente entre 1991 e 1995, respondendo por 28,9% dos cânceres do sexo feminino, percentual similar ao observado entre 2010 e 2014 (29,6%).

A taxa de detecção padronizada por idade do carcinoma *in situ* da mama aumentou de 1,70 no primeiro período para 8,91 por 100 mil mulheres no segundo, atingindo uma razão de taxa de 5,23 (IC95% 4,98–5,50). A taxa de incidência do tumor invasivo também aumentou de 50,15 para 70,03 por 100 mil mulheres, com uma razão de taxas de 1,40 (IC95% 1,33–1,47), enquanto a taxa de mortalidade apresentou redução entre os períodos analisados, passando de 16,18 para 13,79 por 100 mil mulheres, com razão de taxas de 0,85 (IC95% 0,82–0,89) (Tabela 2).

**Tabela 2:** Incidência e mortalidade de câncer de mama (taxas por 100 mil mulheres), Campinas, 1991–1995 e 2010–2014.

Taxas	1991–1995 (1)	2010 –2014 (2)	Razão entre taxas (2/1)
Taxa bruta de detecção do câncer *in situ*	1,52 (1,01–2,04)^a^	11,04 (9,82–12,26)^a^	7,25
Taxa padronizada^b^ de detecção do câncer *in situ*	1,70 (1,16–2,24)^a^	8,91 (8,61–9,21)^a^	5,23 (4,98–5,50)^a^
Taxa bruta de incidência do câncer invasivo	44,51 (41,74–47,28)^a^	88,92 (85,44–92,38)^a^	2,01
Taxa padronizada^b^ de incidência do câncer invasivo	50,15 (46,98–53,31)^a^	70,03 (67,27–72,79)^a^	1,40 (1,33–1,47)^a^
Taxa bruta de mortalidade	13,94 (12,39–15,49)^a^	18,30 (16,73–19,87)^a^	1,31
Taxa padronizada^b^ de mortalidade	16,18 (15,67–16,69)^a^	13,79 (13,46–14,12)^a^	0,85 (0,82–0,89)^a^

^a^ Intervalo de confiança de 95%.

^b^ Taxas padronizadas por idade pela população-padrão mundial de 1960, modificada por Doll et al^17^.

A proporção de casos de CM *in situ* em relação ao total dos cânceres de mama diagnosticados foi de 3,31% no período de 1991–1995 e de 11,05% no período de 2010–2014.

## DISCUSSÃO

Os resultados deste estudo evidenciaram, entre os períodos analisados, aumento de 3 vezes na taxa de detecção do CCU *in situ* e de 5,2 vezes para o CM *in situ*, bem como redução das taxas de mortalidade tanto do CM (declínio de 15%) como do CCU (58%). Entretanto, enquanto a taxa de tumor invasivo declina de forma importante (57%) para o CCU, observa-se um aumento de 40% para o CM invasivo.

Tendência de queda das taxas de incidência e de mortalidade do CCU tem sido observada na maioria dos países nas últimas décadas^3,4^. Análise com dados dos RCBP de dez capitais brasileiras em diferentes períodos, variando de 1996 a 2011, mostrou redução da incidência do CCU invasivo na maioria dos municípios, principalmente em São Paulo, Goiânia e Curitiba, enquanto o CCU *in situ* apresentou tendência de aumento em todas as localidades estudadas^12^. No município de Aracaju, no período de 1996 a 2015, a taxa de incidência do CCU *in situ* passou de 1,19 para 30,2 por 100 mil mulheres, enquanto a do CCU invasivo apresentou queda, passando de 32,3 para 9,1 por 100 mil mulheres^13^. Estudo realizado na região de Barretos (estado de São Paulo) detectou, entre 2000 e 2015, importante crescimento da incidência do CCU *in situ*, que passou de 10,9 para 24,84 por 100 mil mulheres, enquanto a incidência do CCU invasivo ficou estável^11^.

A redução da mortalidade por CCU encontrada em Campinas também foi observada em outros estudos realizados no Brasil, mas as tendências diferem entre regiões e estados em diferentes períodos^18,19^. Estudo que analisou a tendência da mortalidade por CCU no Brasil em três décadas, de 1980 a 2010, mostrou queda em todo o período, exceto nos municípios do interior dos estados das regiões Norte e Nordeste^18^. Essa queda provavelmente reflete a ampliação do acesso ao exame de Papanicolau, que pode não ter ocorrido em municípios do interior das regiões Norte e Nordeste. Vale et al., analisando a tendência da mortalidade por CCU entre 2003 e 2012, verificaram que as taxas só não diminuíram na região Norte, e que os maiores declínios foram observados nas regiões Centro-Oeste e Sul^19^. Redução importante na mortalidade por CCU (36%) também foi verificada no município de Aracajú, onde a taxa de mortalidade passou de 8,1 em 1996 para 5,2 por 100 mil mulheres em 2015^13^.

Em relação ao câncer de mama, em Campinas houve aumento da detecção do CM *in situ* (524%) e da taxa de incidência do CM invasivo (40%), entre os períodos analisados. Essa tendência de aumento também foi constatada na região de Barretos entre 2000 e 2015, com a taxa de incidência do tumor *in situ* passando de 1,6 para 16, e do CM invasivo de 29,1 a 64,1 por 100 mil mulheres^11^.

O aumento da incidência do CM observado em Campinas e na região de Barretos também ocorre mundialmente. Os maiores aumentos nos últimos anos foram detectados em países da América do Sul, da África e da Ásia^1^. Na Europa, América do Norte e Austrália, a incidência aumentou nas décadas de 1980 até meados de 1990, quando se estabilizou, começando a declinar a partir dos anos 2000^1,3^. São apontadas como causas do aumento verificado na década de 1980: melhora no rastreamento do câncer de mama, aumento da prevalência de fatores de risco relacionados ao estilo de vida e terapia de reposição hormonal^3,20^. Não existe uma tendência uniforme na incidência do CM no Brasil. Capitais como Aracaju, Goiânia e Belo Horizonte apresentam tendência de aumento nas últimas décadas, enquanto em São Paulo e Curitiba a tendência é de redução^21^.

A taxa de mortalidade por CM em Campinas não acompanhou o aumento da incidência, apresentando queda entre os períodos estudados, diferentemente do ocorrido em outras localidades do Brasil, mas em consonância com o observado em cidades do Sul e do Sudeste brasileiro^18,21^. A mortalidade por câncer de mama começa a cair a partir dos anos 1990 nas capitais das regiões Sul e Sudeste, mas aumenta nas demais regiões^21^. A diferença deve estar relacionada ao acesso a exames de mamografia e ao aumento da incidência em algumas regiões.

O aumento da detecção do CCU *in situ* em Campinas provavelmente se deve à estratégia de rastreamento implantada no município desde a década de 1960^22^. Já a redução da incidência do CCU invasivo pode ser atribuída ao aumento do diagnóstico do CCU *in situ*, à disponibilidade de rede de atenção para tratamento das lesões precursoras e ao seguimento das pacientes. A região de Campinas foi pioneira na implantação do exame citológico para diagnóstico do CCU, por meio de parceria entre a Universidade Estadual de Campinas e as secretarias municipais e estaduais de saúde. A descentralização da coleta da citologia oncótica, que passou a ser feita nas UBS, ampliou o acesso das mulheres ao diagnóstico precoce do CCU. A redução da mortalidade no período do estudo deve estar relacionada à queda da incidência do CCU invasivo e ao aumento da detecção em estágios mais precoces, possibilitando tratamentos mais oportunos, que aumentam o potencial de cura^23^.

O aumento da detecção do CM *in situ* pode ser consequência do maior acesso à mamografia^24,25^. É preciso considerar, porém, que tal aumento talvez reflita algum grau de sobrediagnóstico, ou seja, a detecção de tumores indolentes que são detectados através da mamografia que não se manifestariam clinicamente. Cálculos de incidência em excesso a partir de auditorias de programas de larga escala estimam que cerca de 20% até 50% dos casos *in situ* ou invasivos detectados através do rastreamento podem representar sobrediagnóstico^26^. É desejável que a proporção de casos *in situ* seja de até 15% do total de tumores diagnosticados^27^. No presente estudo, o número de CM *in situ* correspondeu a 3,31% no período 1991–1995 e 11,05% no período 2010–2014, faixa inferior, portanto, à considerada indicadora de sobrediagnóstico.

O aumento da taxa de incidência do CM invasivo entre os períodos analisados pode refletir a maior exposição das mulheres a fatores de risco ligados a hábitos de vida, como obesidade, primeira gravidez depois dos 30 anos, diminuição do número de filhos e sedentarismo, entre outros, assim como maior acesso à terapia de reposição hormonal^20^. Diagnóstico em estágios mais precoces e melhores tratamentos podem ter contribuído para a redução da mortalidade por CM observada neste estudo^25^.

A cobertura dos exames Papanicolaou e de mamografia em Campinas tem aumentado nos últimos anos. Dados do Inquérito de Saúde de Base Populacional de Campinas (Isacamp) mostram aumento da cobertura de Papanicolaou entre mulheres de 40 a 59 anos de 80,6% para 87,8% entre 2002 e 2008^24,28^. A realização de mamografia entre mulheres com 40 anos ou mais nos dois anos anteriores à entrevista passou de 52,1% em 2002 para 64,2% em 2008^24^. Vale ressaltar que esses resultados se referem à realização de mamografia nos últimos dois anos e de exame de Papanicolaou nos últimos três anos a partir de informação autorreferida pelas entrevistadas.

Estudo que analisou as informações dos Registros Hospitalares de Câncer no estado de São Paulo entre 2000 e 2015 mostrou tendência de aumento no diagnóstico de tumores de mama localizados e nos estágios iniciais da doença, o que pode explicar o aumento da incidência do CM *in situ* e invasivo e a queda da mortalidade por câncer de mama observada em Campinas^25^.

O município de Campinas tem uma rede básica de saúde estruturada desde os anos 1970. Essa rede foi ampliada ao longo das útimas décadas e atualmente conta com 66 UBS. A atenção básica, ordenadora da assistência e coordenadora do cuidado, exerce papel primordial nas ações de promoção, prevenção e planejamento do cuidado para os casos suspeitos e diagnosticados de câncer no território sob sua responsabilidade^9^. Em 2015, Campinas tinha uma cobertura de atenção básica de aproximadamente 50%. Nos anos seguintes, essa abrangência diminuiu, de modo que em 2019 a cidade contava com 128 equipes de saúde da família e uma cobertura de 36,5% da população^29^.

É preciso considerar que o presente estudo utiliza dados dos primeiros anos do RCBP de Campinas, em seus dois momentos de implantação, 1991 e 2010. No entanto, as informações do RCBP para o período de 1991–1995 fizeram parte da publicação da (órgão da Organização Mundial da Saúde) no documento *Cancer incidence in five continents: volume VIII*^30^, publicado em 2002, o que atesta sua qualidade. E no período de 2010–2014 os indicadores de cobertura do RCBP estão de acordo com a recomendação da Iarc^31^.

São poucos os estudos sobre incidência de câncer no Brasil, já que os dados estão disponíveis apenas para municípios que possuem RCBP ativos. Em 2019, havia 33 RCBP no Brasil, dos quais apenas 27 estavam ativos^2^. Além disso, é preciso que os dados cubram um longo período para que mudanças sejam analisadas, o que foi possível no presente estudo, já que os dois períodos considerados estão separados por 20 anos.

Para os programas de rastreamento serem efetivos é importante organizar a rede à saúde, tanto na atenção básica quanto nas unidades de referência para tratamento. O Planejamento Estratégico do Ministério da Saúde para o período 2011–2015 definiu ações do Governo Federal para qualificar o programa de mamografia e ampliar serviços de diagnóstico do CM e do CCU^32^. No entanto, além de garantir acesso a práticas preventivas (o que foi propiciado pelo expressivo crescimento e fortalecimento da atenção básica), também é essencial que o tratamento seja garantido em tempo oportuno.

## CONCLUSÕES

Em quase duas décadas, as taxas de incidência de carcinomas *in situ* de CCU e CM aumentaram de modo significativo (mais de três e sete vezes, respectivamente). A taxa de carcinoma invasivo do CCU diminuiu, mas a de CM aumentou. Um resultado positivo encontrado pelo presente estudo foi a redução das taxas de mortalidade por CM e a persistência da redução de mortes por CCU. Observar essas alterações ajuda a avaliar o impacto das ações realizadas no período e planejar ações futuras.
